# Metabolic effects of CCL5 deficiency in lean and obese mice

**DOI:** 10.3389/fimmu.2022.1059687

**Published:** 2023-01-13

**Authors:** Hui Zhou, Xiyan Liao, Qin Zeng, Haowei Zhang, Jianfeng Song, Wanyu Hu, Xiaoxiao Sun, Yujin Ding, Dandan Wang, Yalun Xiao, Tuo Deng

**Affiliations:** ^1^ National Clinical Research Center for Metabolic Diseases, and Department of Metabolism and Endocrinology, The Second Xiangya Hospital of Central South University, Changsha, China; ^2^ Key Laboratory of Diabetes Immunology, Ministry of Education, and Metabolic Syndrome Research Center, The Second Xiangya Hospital of Central South University, Changsha, China; ^3^ Clinical Immunology Center, The Second Xiangya Hospital of Central South University, Changsha, China

**Keywords:** obesity, adipose inflammation, CCL5, chemokine, insulin resistance

## Abstract

Accumulation and activation of immunocytes in adipose tissues are essential to obesity-induced inflammation and insulin resistance. Chemokines are pivotal for the recruitment of immunocytes in adipose tissue during obesity. Chemokine (C-C motif) ligand 5 (CCL5) plays a vital role in the recruitment of immunocytes to sites of inflammation. CCL5 expression level is increased in obese adipose tissue from humans and mice. However, the role of CCL5 in obesity-induced adipose inflammation remains unclear. Our study found that the CCL5 expression level was increased in the epididymal white adipose tissue (eWAT) of obese mice, particularly in CD8^+^ T cells. CCL5 knockout (KO) mice exhibited better glucose tolerance than wild-type (WT) mice under lean conditions. In contrast, CCL5 KO mice were more insulin resistant and had severe hepatic steatosis than WT mice under obese conditions. Increased T cells in adipose tissue heaven adipose inflammation in obese CCL5 KO mice. The compensatory increased T cell-associated chemokines may account for increased T cell content in the eWAT of obese CCL5 KO mice. These findings imply that CCL5 deficiency exacerbates adipose inflammation and impairs insulin sensitivity in the metabolic tissues of obese mice.

## 1 Introduction

Obesity is a major public health issue worldwide. The prevalence of obesity increased from 0.7% (95% credible interval [CrI] 0.4-1.2) in 1975 to 5.6% (4.8-6.5) in 2016 for girls aged 5-19 years, and from 0.9% (0.5-1.3) to 7.8% (6.7-9.1) for boys (1). Obesity is a state of chronic inflammation and leads to many complications such as type 2 diabetes, cardiovascular disease, and cancer (2, 3). Chronic inflammation contributes to the development of insulin resistance and glucose intolerance. Chronic inflammation is induced by obesity in many tissues, including adipose tissue, liver, muscle, gastrointestinal tract, central nervous system, and pancreatic islets (4). Adipose inflammation is a major contributor to insulin resistance in obesity (5). Several potential mechanisms, including gut-derived antigens, dietary or endogenous lipids, adipocyte death, hypoxia, and mechanical stress, have been reported to participate in obesity-induced adipose inflammation (6). Many studies have demonstrated that the increased accumulation of proinflammatory immunocytes plays a central role in obesity-induced adipose inflammation (4, 7, 8). Notably, macrophages and T cells are the dominant immune cell types accumulated in obese adipose tissue (4). Both inflammatory macrophages (M1-like macrophages) and T cells (Th1 and CD8^+^ T cells) are increased in adipose tissue from obese patients and mice (2, 4). Therefore, understanding the mechanisms underlying obesity-induced immunocyte accumulation in adipose tissue will provide new insights into treating obesity-induced adipose inflammation and complications.

Chemokines are small proteins that direct the infiltration of circulating leukocytes to the sites of inflammation or injury (9). C-C motif chemokine ligand 5 (CCL5, also known as RANTES) is a chemokine that could recruit leukocytes to inflammatory tissues (10). CCL5 gene expression was elevated in adipose tissue from obese patients and mice (11, 12). Moreover, the mRNA expression level of CCL5 was positively correlated with T cell marker CD3 and macrophage marker CD11b in the visceral adipose tissue of obese patients (12, 13). CCL5 could be secreted by various cell types, including T cells, macrophages, epithelial cells, fibroblasts, and platelets (10, 14). The mRNA expression level of CCL5 was induced by obesity more markedly in the stromal vascular fraction (SVF) than in adipocytes (15). However, the major cellular source of CCL5 in obese adipose tissue remains unclear.

CCL5 regulates the trafficking and homing of various immunocytes, including T cells, monocytes, granulocytes, and eosinophils (10). It binds to at least four receptors, including CCR1, CCR3, CCR5, and GPR75 (16, 17). Several groups have attempted to identify the role of CCR5, a major receptor of CCL5 in adipose tissue, in obesity-induced insulin resistance. Kitade et al. found that CCR5-deficient mice exhibited improved insulin sensitivity and glucose tolerance due to decreased macrophage accumulation in obese adipose tissue (18). However, Kennedy et al. illustrated that CCR5 deficiency exacerbated glucose tolerance and increased CD4^+^ T cells but not macrophage infiltration into adipose tissue in obesity (19). Since CCR5 can bind to other ligands, including CCL3, CCL4, CCL8, and CCL14, CCR5 deficient mouse is not an ideal animal model to investigate the role of CCL5 in adipose inflammation. Therefore, the role of CCL5 in obesity-induced adipose inflammation and insulin resistance is obscure.

In this study, we identified the major cellular source of CCL5 in obese mice and employed CCL5 knockout (KO) mice to determine the role of CCL5 in obesity-induced adipose inflammation and insulin resistance. We found that the expression level of CCL5 was increased in obese epididymal white adipose tissue (eWAT), particularly in the CD8^+^ T cells. CCL5 deficiency enhanced glucose tolerance in lean mice but exacerbated insulin resistance and adipose inflammation in obesity. CCL5 deficiency leads to increased T cells accumulation in obese adipose tissue, possibly due to compensatory upregulation of other chemokines.

## 2 Material and methods

### 2.1 Mice

C57BL/6J mice were purchased from Slac Laboratory Animal Inc (Shanghai, China), and CCL5 KO mice were purchased from Jackson Laboratory (Stock NO. 005090, Bar Harbor, ME, USA). All mice were kept in the specific pathogen-free animal room, maintaining a constant temperature and 12h/12h light/dark cycle. All animal procedures followed the Care and Use of Laboratory guidelines at Central South University. The C57BL/6J and CCL5 KO mice were crossed, and the F1 CCL5 heterozygous mice were used to generate wild-type (WT) and CCL5 KO littermates for experimental research. At 6 weeks of age, male mice were fed a normal diet (ND,10% fat, MD17121) (Medicience Ltd, Jiangsu, China) or a high fat diet (HFD; 60% fat, D12492) (Research Diets, New Brunswick, NJ). At 22 weeks of age, four cohorts of mice were euthanized. Tissues were collected for subsequent experiments.

### 2.2 Adipose tissue SVF isolation

EWAT were excised and minced in a 5 ml centrifuge tube. 1mg/ml type II collagenase (Worthington Biochemical, NJ, USA) in PBS containing 1% BSA was added to the minced adipose tissues and digested for 30 min at 37°C with shaking. The cell suspension was filtered through a 70 μm filter and centrifuged at 500*g* for 5 min to separate the adipocytes from the SVF pellet. Following centrifugation, the SVF pellet was suspended in erythrocyte lysate and incubated on ice for 5 min to lyse red blood cells. Cells were used for flow cytometry.

### 2.3 Flow cytometry

For surface markers detection, SVF cells were suspended in PBS containing 1% fetal bovine serum and incubated with Zombie dye (1:100, Cat#423106, Biolegend, USA) at room temperature for 7 min. Then cell suspension was incubated with CD16/32 (1:100, Cat#101302, Biolegend, USA) at room temperature for 7 min, followed by incubation with fluorochrome-conjugated antibodies (**Supplementary Table 1**) for 7 min at room temperature. For intracellular staining, samples were added with Monensin (1:1000, Cat#420701, Biolegend, USA) in the whole process to block the secretion of CCL5. The SVF cells were incubated with Zombie dye, blocked by CD16/32, and stained with fluorochrome-conjugated antibodies (**Supplementary Table 1**) against cell surface antigens. Then cells were fixed and permeabilized with cytofix/cytoperm buffer (Cat#554714, BD Bioscience, USA) for 45 min at 4 °C and then stained with fluorochrome-conjugated antibody against CCL5 for 30 min at 4 °C. Cells were washed by PBS twice and centrifuged at 500*g* for 5 min, and the cell pellet was suspended in 200 μL FACS Buffer. Samples were processed on a CYTEK flow cytometer and analyzed using FlowJo software.

### 2.4 Glucose tolerance test

Mice were fasted for 16 h. After baseline blood glucose collection, 20% glucose solution (1 g/kg) was administered intraperitoneally, and tail vein blood glucose was measured at 15, 30, 45, 60, and 120 min.

### 2.5 Insulin tolerance test

Mice were fasted for 6 h. After baseline blood glucose collection, 0.45 U/kg (ND group) or 0.75 U/kg (HFD group) insulin was administered intraperitoneally, and tail vein blood glucose was measured at 15, 30, 45, 60, and 90 min.

### 2.6 Serum insulin and GLP-1 level measurements

Mice were fasted for 6 h, and orbital venous plexus blood was collected to detect basal fasting insulin levels. After fasted overnight, 20% glucose solution (1 g/kg) was administered intraperitoneally, and orbital venous plexus blood was collected to detect glucose-stimulated insulin levels. Blood samples were centrifuged at 3000 g for 15 min, and serum insulin concentration was detected using an insulin Elisa kit (Cat# 32270, IMD, Hongkong, China).

Mice were fasted for 6 h. After basal blood samples were taken, 2 g/kg glucose was administered by gavage, and tail vein blood was collected after 15 min. Serum GLP-1 level was measured by ELISA kit (Cat#AF2027-A, AiFang, Changsha, China). Absorbance at 450 nm was determined using a microplate reader.

### 2.7 Staining of tissue sections

EWAT, liver, pancreas, and intestinal were harvested and fixed overnight in 4% paraformaldehyde, paraffin-embedded, then sectioned (5 μm), followed by hematoxylin and eosin (H&E) staining. Adipose tissue sections were hybridized with CD3 (Cat#AF20162, AiFang, Changsha, China) and F4/80 (Cat#SAF002, AiFang, Changsha, China) antibodies. Alexa Flour 488 donkey anti-mouse IgG (Cat# A32766, Invitrogen, USA) was used as a secondary antibody to detect CD3^+^ cells. Alexa Fluor 594 goat anti-rabbit IgG (Cat#A32740, Invitrogen, USA) was used as a secondary antibody to detect F4/80^+^ cells. For the detection of macrophage apoptosis, the TUNEL assay was performed using a FITC TUNEL cell apoptosis detection kit (Cat# G1501-100T, Servicebio, Wuhan, China).

Pancreatic paraffin-embedded tissue sections were stained with mouse anti-insulin (Cat# 66198-1, Proteintech, USA), rabbit anti-Glucagon (Cat#ab92517, Abcam, The UK), rabbit anti-Ki67 (Cat# D3B5, Cell Signaling Technology, USA) antibodies. Alexa Flour 488 donkey anti-mouse IgG (Cat# A32766, Invitrogen, USA) and Alexa Fluor 594 goat anti-rabbit IgG (Cat# A32740, Invitrogen, USA) were used as secondary antibodies. Intestinal paraffin-embedded tissue sections were stained with mouse anti-GLP1 (Cat# sc-514592, Santa Cruz, USA). Images were acquired with an Olympus microscope and integrated density was analyzed with Image J Software.

### 2.8 Cell proliferation assay

Mouse insulinoma cells (MIN6) were cultured in a 96-well plate at a density of 3.0 ×10^3^ cells per well. GLP-1 (100 nM) with or without CCL5 (100 ng/ml) was mixed into the cell cultures. After 0, 24, 48, and 72 h of incubation, 10 μL CCK-8 solution was mixed into the culture and further incubated for 2 hours. Cell viability was measured at a 450 nm wavelength (OD450). The cell viability ratio (CRV) was calculated as (A-A_0_)/A_0_ × 100% (A was the absorbance of the treated cell culture and A_0_ was the OD450 value of a blank (DMEM medium only).

### 2.9 Primary hepatocyte isolation

Mouse primary hepatocytes were isolated following a 2-step collagenase digestion protocol (20). Briefly, mice were anesthetized and the liver was perfused *in situ* with 50 ml Hank’s Balanced Salt Solution (HBSS) through the portal vein, followed by 8 mL of liver digestion media containing 2M HEPES, 1% Penicillin-Streptomycin (P/S) Solution, and 0.08% type 4 collagenase. The liver was excised, minced, and filtered through a 100-micron mesh. The isolated hepatocytes were centrifuged at 50g for 3 min, and then the cell pellet was suspended in the MEM-α medium containing 80 μg/L DEX, 10% FBS, and 1% P/S Solution.

### 2.10 Primary skeletal muscle cell isolation

Skeletal muscle was harvested and washed by PBS three times and then minced with scissors. Skeletal muscle fragments were digested in the muscle digestion media containing 0.1% Pancreatin and 1mg/mL type 2 collagenase at 37°C for 25 min. After passing cells through a 70 μm cell strainer and centrifugation at 1000 rpm for 10 min, the cell pellet was suspended in DMEM/F12 medium containing 1% P/S, and 20% FBS. The fibroblasts were removed by differential adherence.

### 2.11 *In vitro* adipocyte differentiation

SVF from iWAT of C57BL/6J mice were cultured in DMEM/F12 plus 10% FBS, 1% Pen/Strap, and b-FGF (10ng/ml, Cat#100-18B, Peprotech, USA). Cells were allowed to grow to confluence and treated with white adipocyte differentiation induction cocktail: 0.5 mM 3-isobutyl-1-methylxanthine (IBMX, Cat# 15879-1G, Sigma, USA), 1 uM dexamethasone (Cat# D4902, Sigma, USA), 1.7 μM insulin, followed by maintenance treatment (1.7 μM insulin) until day 7-8 for harvest.

### 2.12 Insulin signaling

Cells were cultured in MEMα or DEME containing 4% FBS and treated with or without CCL5 (100 ng/ml) for 12 h, then stimulated with 100 nM insulin for 15 min. After stimulation, cells were washed immediately with PBS before lysis and scraped down in RIPA buffer containing protease and phosphatase inhibitors. Then, western blot analysis was performed.

### 2.13 *In vitro* chemotaxis assay

C57BL/6J mice thioglycolate-elicited peritoneal macrophages were isolated. For the migration *per se*, 1 ×10^5^ intraperitoneal macrophages were placed in the upper chamber of an 8 μM polycarbonate filter (12-transwell format; Corning, Lowell, MA), RPMI 1640 medium with or without CCL5 (10 ng/ml) was placed in the lower chamber. After 1 h of migration, the upper layer and trans-well insert were carefully removed. Migrated macrophages were counted using a cell counter chamber.

### 2.14 Macrophage apoptosis assays

RAW264.7 were incubated with LPS (1 μg/ml) or palmitate (0.3 mM) and treated with or without CCL5 (100 ng/ml) for 16 h. Cells were stained with an annexin V and propidium iodide (PI) double-staining technique and then analyzed using a CYTEK flow cytometer.

### 2.15 Primary hepatocytes lipid treatment

Primary hepatocytes were cultured with 10% FFA-free BSA-conjugated fatty acid (0.4 mM oleic acid and 0.2 mM palmitate) with or without CCL5 (100 ng/ml) for 24 h for RNA extraction and oil red O experiments. The stained cells were photographed with an Olympus microscope, After the dye retained in the cells was extracted with isopropanol, the OD510 was determined using a microplate reader.

### 2.16 Western blots analysis

After 16 weeks of HFD, Mice were fasted for 6 h and then intraperitoneally administered with PBS or insulin (4 U/Kg). Mice subsequently were euthanized 15 min later, and eWAT, liver, skeletal muscle were collected, frozen in liquid nitrogen immediately, and stored at −80°C. Total protein was isolated by RIPA buffer (Beyotime, Shanghai, China) containing protease (Roche, Basel, Switzerland) and phosphatase inhibitors (Roche, Basel, Switzerland). Protein concentration was measured with a BCA kit (Dingguochangsheng, Beijing, China) and the same amount of total protein was loaded onto polyacrylamide gels. Proteins were isolated and then transferred to PVDF membranes. Membranes were blocked for 1 h in 5% BSA at room temperature. The membranes were first incubated with following anti-phosphotyrosine AKT Ser473 (1:1000, Cat#4060, Cell Signaling Technology, USA) antibody at 4°C overnight. The membranes were subsequently stripped using solution containing 62.5 mM Tris-HCl, 2%SDS, 100 mM b-mercaptoethanol at 55°C for 25 min and reincubated with anti-AKT (1:1000, Cat#9272, Cell Signaling Technology, USA) antibody at 4°C overnight. Integrated density was analyzed with Image J Software.

### 2.17 RNA isolation and real-time RT-PCR

Total RNA was extracted using Trizol reagent (Invitrogen, USA) and 1000 ng RNA was reversed using cDNA Synthesis Kit (Thermo-Fisher Scientific, MA, USA). The synthesized cDNA was diluted 5 times in enzyme-free water. qRT-PCR was performed on a real-time fluorescence quantizer (ABI, USA), and all qRT-PCR primer sequences are shown in **Supplementary Table 2**. After normalization with housing-keeping gene β-actin or 36B4 mRNA, the relative expression levels of target genes were calculated by the ΔΔCT method.

### 2.18 Oil red O staining

Liver tissue was fixed in 4% paraformaldehyde overnight, embedded in OCT glue, and frozen at −80°C for over 24 h. The embedded tissue was removed and sectioned in a frozen slicer at −20°C. Frozen sections were placed at room temperature for 10 minutes and washed with PBS 3 times. The sections were subsequently washed in 60% isopropyl alcohol for 5 min and stained with 60% Oil red O working solution for 15 min away from light. The sections were rinsed in 60% isopropyl alcohol to allow the staining of fat cells to bright red and other cells to be colorless, excess dye and isopropyl alcohol were washed under running water. The nucleus was stained with hematoxylin for 3 min, and the slides were then sealed with glycerin gelatin.

### 2.19 Statistical analysis

All data were processed by SPSS V19.0 statistical software. The statistical results were presented as means ± SEM, and comparison was performed by Student’s *t-*test or two-way ANOVA. *p*<0.05 indicated that the difference was statistically significant (^+^ *p <*0.1, *p<0.05, **p<0.01, ***p<0.001).

## 3 Results

### 3.1 CCL5 and its receptors are increased in CD8^+^ T cells from eWAT of obese mice

**Figure 1:**
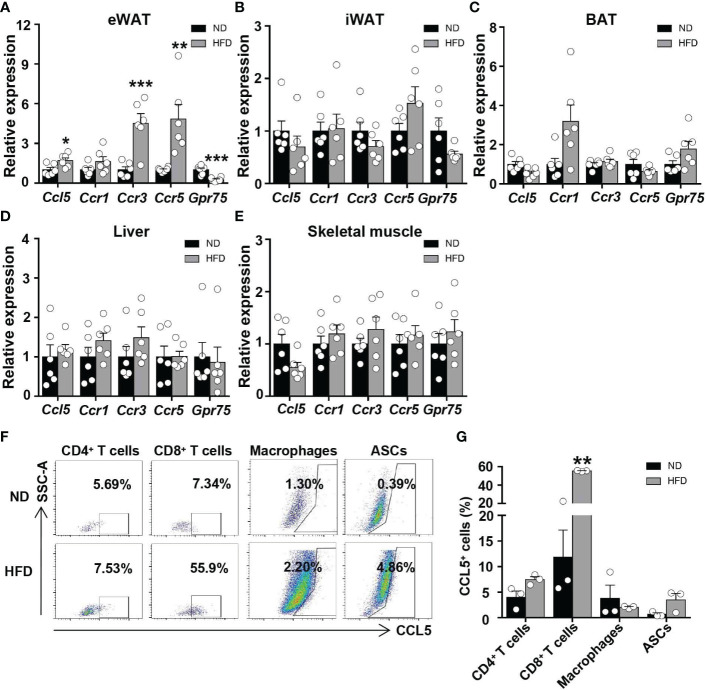
CCL5 is upregulated in CD8^+^ T cell in eWAT of obese mice. **(A-E)** mRNA expression of CCL5 and its receptors in eWAT **(A)**, iWAT **(B)**, BAT **(C)**, liver **(D)** and skeletal muscle **(E)** in ND-fed and 16-week HFD-fed mice. (n = 6 mice per group). **(F)** Representative flow cytometry plots of CCL5 expression in CD4^+^ T cells, CD8^+^ T cells, macrophages, and ASCs of ND-fed and 16-week HFD-fed mice. **(G)** Quantification of CCL5^+^ cells in CD4^+^ T cells, CD8^+^ T cells, Macrophages, and ASCs in C57BL/6J mice fed the ND or HFD by flow cytometry analysis. (n = 3 mice per group). Data are mean ± s.e.m. *p<0.05, **p<0.01, ***p<0.001 by unpaired Student’s *t*-test.

To investigate the regulation of CCL5 expression by obesity in metabolic tissues, we analyzed mRNA levels of CCL5 and its receptors in adipose tissues, liver, and skeletal muscle from ND and HFD-fed mice. The mRNA levels of CCL5 and its receptors, CCR3 and CCR5, were significantly increased in the eWAT of obese mice compared with lean mice (**Figure 1A**). The mRNA levels of CCL5 and its receptors did not differ between lean and obese mice in inguinal white adipose tissue (iWAT), brown adipose tissue (BAT), liver, and skeletal muscle (**Figures 1B–E**). Wu et al. demonstrated that the mRNA level of CCL5 was markedly higher in SVF than in adipocytes of eWAT from obese mice (12). SVF is composed of various cells, including immunocytes and adipose stem cells. To identify the dominant cellular sources responsible for the upregulation of CCL5 in eWAT by obesity, we detected the expression of CCL5 in CD4^+^ T cells, CD8^+^ T cells, macrophages, and adipose tissue stem cells (ASCs) in eWAT from lean and obese mice by flow cytometry. The gating strategy was shown in **Supplementary Figure 1**. The percentage of CCL5 positive CD8^+^ T cells was significantly increased in obese mice compared with lean mice (**Figures 1F, G**). It was noticeable that CD8^+^ T cells had the highest proportion of CCL5 positive cells in SVF, and up to 60% of CD8^+^ T cells are CCL5 positive cells in eWAT from obese mice (**Figure 1G**). To investigate whether CCL5 is specifically increased in CD8^+^ T cells in adipose tissues during obesity, we examined the percentage of CCL5 positive CD8^+^ T cells in peripheral blood, mesenteric lymph nodes, and spleen of lean and obese mice. The percentages of CCL5 positive CD8^+^ T cells were not changed in blood and spleen, and increased mildly in mesenteric lymph nodes, suggesting that the expression of CCL5 in CD8^+^ T cells is tissue-specifically increased in adipose tissue of obese mice (**Figures S2A**). We also examined the cellular subtypes of CCL5-expressing CD8^+^ T cells in adipose tissue. The results showed that effector memory CD8^+^ T cells are the major subtype of CCL5 positive CD8^+^ T cells (**Figures S2B, C**). Collectively, these data demonstrate that CCL5 expression levels are increased in the eWAT of obese mice, particularly in adipose tissue CD8^+^ T cells.

### 3.2 CCL5 deficiency improves glucose tolerance in chow diet-fed mice

**Figure 2:**
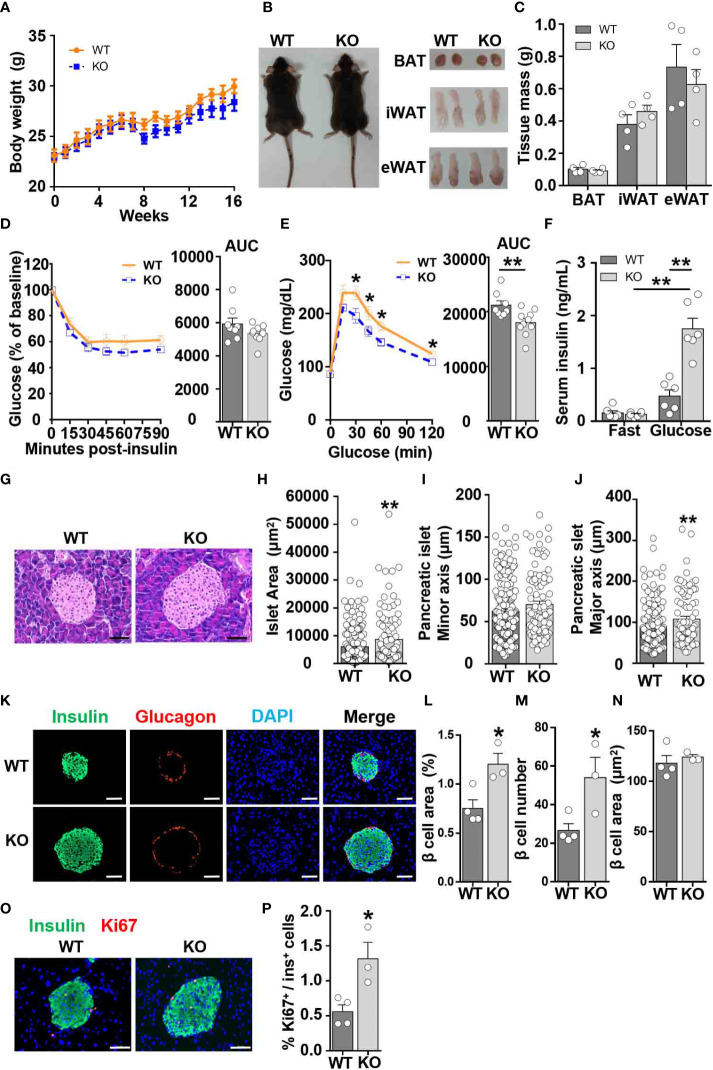
CCL5 deficiency enhances glucose tolerance in ND-fed mice. **(A)** Body weights of WT and CCL5 KO mice fed an ND. (n = 4-6 mice per group). **(B)** Representative images of mice and each part of fat pad (BAT, iWAT, and eWAT). **(C)** Weight of BAT, iWAT, and eWAT of WT and CCL5 KO mice fed an ND. (n = 4-6 mice per group). **(D, E)** ITT **(D)** and GTT **(E)** in mice fed an ND (n = 8-10 mice per group). **(F)** Serum insulin levels of ND-fed mice after 6 h fasting (fast) or 15 min after 1g/kg glucose injection (glucose). (n = 6 mice per group). **(G)** Representative H&E staining of pancreatic islet section. Scale bar: 50 μm. **(H–J)** area **(H)**, minor axis **(I)**, and major axis **(J)** of each pancreatic islet. (n = 4 mice per group. Data shown in panels **(H–J)** were obtained from the analysis of 16 sections). **(K)** Representative immunofluorescent staining of the islets of mice fed an ND using anti-insulin (green) and anti-glucagon (red) antibodies. **(L)** β cell mass of WT and CCL5 KO mice fed an ND. (n = 3-4 mice per group). **(M)** β cell number per islet of WT and CCL5 KO mice fed an ND. (n = 3-4 mice per group). **(N)** The size of individual β cell in WT and CCL5 KO mice fed an ND. (n = 3-4 mice per group). **(O)** Representative immunofluorescent staining of the islets of mice fed the ND using anti-insulin (green) and anti-ki67 (red) antibodies. Arrows indicate insulin^+^Ki67^+^ cells. Scale bar: 50 μm. **(P)** Percentages of insulin^+^Ki67^+^ cells in pancreatic sections of WT and CCL5 KO mice fed an ND. (n = 3-4 mice per group). Data in **(C–E, H–J, L–N, P)** are mean ± s.e.m. *p<0.05, **p<0.01 by unpaired Student’s t-test. Data in **(F)** is mean ± s.e.m.*p<0.05, **p<0.01 by two-way ANOVA.

To determine whether CCL5 affects systemic metabolism under lean conditions, we examined body weight, insulin sensitivity, and glucose tolerance in WT and CCL5 KO littermate mice. WT and CCL5 KO mice fed on chow diet remained equivalent body weight and adipose tissue mass (**Figures 2A–C**). Although CCL5 KO mice had similar insulin sensitivity to WT mice, CCL5 KO mice exhibited better glucose tolerance than WT mice (**Figures 2D, E**), suggesting an enhanced insulin secretion in CCL5 KO mice. Thus, we examined basal fasting and glucose-stimulated insulin levels in the serum of the two groups. The serum insulin levels were increased in CCL5 KO mice compared with WT mice after glucose stimulation (**Figure 2F**), indicating that CCL5 deficiency promoted insulin secretion.

To determine the role of CCL5 in islet development, islet function from lean WT and CCL5 KO mice was evaluated by H&E and immunofluorescence staining. The islet size was larger in the pancreas of CCL5 KO mice than that of WT mice (**Figures 2G, H**). Moreover, a significant increase in the maximum axis but not the minimum axis of each islet was observed in the pancreas of obese CCL5 KO mice (**Figures 2I, J**). Additionally, the total area of beta cells was significantly increased in CCL5 KO mice than in WT mice (**Figures 2K, L**). Since the number but not the size of beta cells was increased in CCL5 KO mice (**Figures 2M, N**), the increased total area of beta cells in CCL5 KO mice is mainly due to the increased beta cell number in the islet. Finally, we examined the proliferation of islet beta cells and observed more Ki67 positive proliferating islet beta cells in CCL5 KO mice than in WT mice (**Figures 2O, P**). It has been shown that administration CCL5 in mice reduces plasma GLP-1 and GLP-2 (21). To investigate whether CCL5 affects islet beta cell proliferation by regulating the expression of GLP-1, we examined serum GLP-1 levels in WT and KO mice during fasting and after glucose gavage. The serum GLP-1 levels were significantly increased in KO mice under both fasting and glucose gavage conditions (**Figure S3A**). In addition, compared with WT mice, the mRNA and protein expression levels of GLP-1 in the jejunum, ileum, and colon of KO mice were also significantly increased (**Figures S3B-D**). These results suggest that CCL5 is an inhibitor the expression of GLP-1. To further investigate whether CCL5 affects the function of GLP-1, we treated min6 cells with or without GLP-1 and CCL5. the results showed that CCL5 inhibited the proliferation of min6 cells and the GLP-1-induced proliferation of min6 cells (**Figure S3E**). Together, these data indicate that CCL5 deficiency enhances glucose tolerance by promoting beta cell proliferation under lean conditions.

### 3.3 CCL5 deficiency promotes HFD-induced insulin resistance

**Figure 3:**
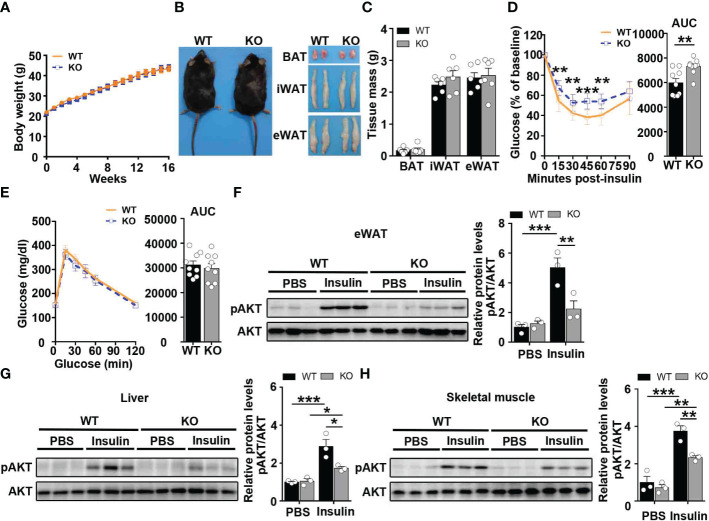
CCL5 deficiency aggravates insulin resistance in HFD-fed mice. **(A)** Body weights of WT and CCL5 KO mice after 16 weeks HFD. (n = 9-11 mice per group). **(B)** Representative images of mice and each part of fat pad (BAT, iWAT, and eWAT). **(C)** Weight of BAT, iWAT, and eWAT of WT and CCL5 KO mice after 16 weeks HFD. (n = 6 mice per group). **(D, E)** ITT **(D)** and GTT **(E)** in mice after 16 weeks HFD. (n = 9 mice per group). **(F-H)** Western blot (left) and quantification (right) of p-AKT and AKT in the eWAT **(F)**, liver **(G)**, and skeletal muscle **(H)** of WT and CCL5 KO mice after 16 weeks HFD. (n = 6 mice per group). Values were normalized to WT-PBS group. Data in **(C)** are mean ± s.e.m.**p<0.01, ***p<0.001 by unpaired Student’s t-test. Data in **(D-E)** are mean ± s.e.m. **p<0.01, ***p<0.001 by two-way repeated-measures ANOVA with *post hoc* test by unpaired Student’s t test. Data in **(F-H)** are mean ± s.e.m.*p<0.05, **p<0.01, ***p<0.001 by two-way ANOVA.

To examine the role of CCL5 in obesity-induced insulin resistance, WT and CCL5 KO littermate mice were fed on HFD for 16 weeks. Although WT and CCL5 KO mice revealed equivalent body weight and WAT mass (**Figures 3A–C**), CCL5 KO mice were significantly more insulin resistance than their WT littermates, while glucose tolerance did not differ by genotype (**Figures 3D, E**). To confirm the insulin resistant phenotype of obese CCL5 KO mice, the insulin responsiveness in eWAT, liver, and muscle was assessed by post-insulin AKT phosphorylation. Consistent with ITT results, the pAkt/Akt ratio was decreased in eWAT, liver, and muscle of CCL5 KO mice, indicating that CCL5 deficiency significantly attenuated insulin signal transduction in classic insulin target organs in obesity (**Figures 3F–H**). To further investigate whether CCL5 directly acts on insulin signaling, we treated adipocytes, primary hepatocytes, and primary skeletal muscle cells with CCL5 and examined its effect on insulin signaling. CCL5 has no direct effect on insulin signaling in these three insulin target cells (**Figures S4A–C**), indicating that there are other indirect mechanisms regulating insulin signaling in obese CCL5 KO mice.

### 3.4 CCL5 deficiency exacerbates HFD-induced adipose inflammation

**Figure 4:**
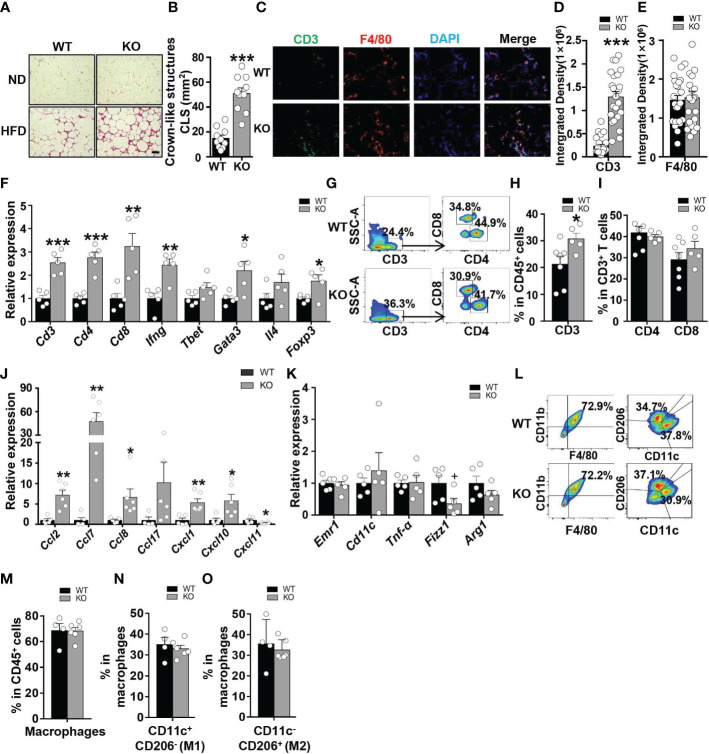
CCL5 deficiency exacerbates HFD-induced adipose inflammation. **(A)** Representative H&E staining of eWAT from WT and CCL5 KO mice after ND-fed and 16-week HFD-fed. Scale bar: 100 μm. **(B)** Quantification of CLSs in WT and CCL5 KO mice after 16 weeks HFD. (n = 10 high power fields from 4 animals in each group). **(C)** Representative immunofluorescent staining of the eWAT from mice fed a HFD using anti-CD3 (green) and anti-F4/80 (red) antibodies. Scale bar: 100 μm. **(D, E)** Quantification of images for CD3 **(D)** and F4/80 **(E)** integrated density in eWAT from WT and CCL5 KO mice after 16 weeks HFD. (n = 25 high power fields from 4 animals in each group). **(F)** mRNA expression of T cell marker genes in eWAT of WT and CCL5 KO mice after 16 weeks HFD. (n = 5 mice per group). **(G)** Representative flow cytometry plots of CD3^+^ T cells, CD4^+^ T cells, and CD8^+^ T cells in eWAT of WT and CCL5 KO mice after 16 weeks HFD. **(H)** Quantification of CD3^+^ T cells in CD45^+^ cells in eWAT of WT and CCL5 KO mice after 16 weeks HFD. (n = 5-7 mice per group). **(I)** Quantification of CD4^+^ T cells, CD8^+^ T cells in CD3^+^ cells in eWAT of WT and CCL5 KO mice after 16 weeks HFD. (n = 5-7 mice per group). **(J)** mRNA expression of chemokine genes in eWAT of WT and CCL5 KO mice fed a HFD. (n=4-6 mice per group). **(K)** mRNA expression of macrophage marker genes in eWAT of WT and CCL5 KO mice after 16 weeks HFD. (n = 5 mice per group). **(L)** Representative flow cytometry plots of macrophages in eWAT of WT and CCL5 KO mice after 16 weeks HFD. CD11c^+^ CD206^-^ (M1-type) and CD11c^-^ CD206^+^ (M2-type). **(M)** Quantification of macrophages in eWAT of WT and CCL5 KO mice after 16 weeks HFD. (n=4-6 mice per group). **(N, O)** Quantification of M1-type **(M)** and M2-type macrophages **(N)** in total macrophages of eWAT from WT and CCL5 KO mice after 16 weeks HFD. (n=4-6 mice per group). Data are mean ± s.e.m. ^+^ *p*<0.1, *p<0.05, **p<0.01, ***p<0.001 by unpaired Student’s t-test.

To investigate whether CCL5 participates in obesity-induced adipose inflammation, we detected immunocyte accumulation in the eWAT of obese WT and CCL5 KO mice. Small and similar numbers of immune cells accumulated in the adipose tissues of lean WT and CCL5 KO mice (**Figure 4A**). However, more immunocytes were observed in the eWAT of obese CCL5 KO mice than in those of obese WT mice (**Figure 4A**). Consistently, obese CCL5 KO mice had more crown-like structures (CLSs) (**Figure 4B**), which are composed of many kinds of immunocytes and serve as an indicator of adipose inflammation (22, 23). Additionally, immunofluorescence staining revealed an increased accumulation of T cells but not macrophages in the eWAT of obese CCL5 KO mice (**Figures 4C–E**).

To confirm the increased T cell accumulation in eWAT of obese CCL5 KO mice, mRNA levels of T cell marker genes in eWAT of WT and CCL5 KO mice were measured by qRT-PCR. The mRNA levels of T cell marker genes were not significantly different between lean WT and CCL5 KO mice (**Figure S5A**). However, T cell marker genes (*Cd3*, *Cd4*, and *Cd8*) as well as Th1 (*Ifn-γ*, *Tbet*/*Tbx21*), Th2 (*Gata3*, *Il4*), and Treg (*Foxp3*) marker genes were all increased in the eWAT of obese CCL5 KO mice (**Figure 4F**). We also analyzed the percentage of T cells in eWAT of obese WT and KO mice by flow cytometry analysis. The percentage of CD3^+^ T cells was significantly increased in the eWAT of obese CCL5 KO mice than that in the eWAT of obese WT mice, and while the percentages of CD4^+^ T cells and CD8^+^ T cells were not different between the two groups (**Figures 4G–I**), indicating that CCL5 deficiency leads to an increase in the entire T cell population rather than a specific T cell subpopulation.

Chen et al. demonstrated that CCL5 deficiency could compensatorily induce the production of other chemokines to enhance immunocyte recruitment (24). To determine whether other chemokines could be induced by CCL5 deficiency, we detected the mRNA expression levels of chemokines that could recruit T cells to tissues. These chemokine expression levels were not increased in the eWAT of lean CCL5 KO mice (**Figure S6A**). However, the mRNA expression levels of CCL2, CCL7, CCL8, CXCL1, and CXCL10 were significantly increased in the eWAT of obese CCL5 KO mice than in WT mice (**Figure 4J**). These data suggest that the increased accumulation of T cells in obese CCL5 KO mice may be mediated by the induction of other chemokines.

Macrophages undergo phenotype switch from an anti-inflammatory M2 phenotype to a proinflammatory M1 phenotype in obesity (4). To determine whether CCL5 regulates the macrophage phenotype switch in adipose tissue, we detected mRNA levels of macrophage marker genes in eWAT of WT and CCL5 KO mice by qRT-PCR. The mRNA levels of M1 marker genes (*Emr1*, *Cd11c*) and M2 macrophage marker genes (*Fizz1* and *Arg1*) showed no significant difference between WT and CCL5 KO mice in both lean and obese conditions (**Figures S5B, 4K**). We further detected the percentage of M1 and M2 macrophages in eWAT of obese WT and CCL5 KO mice by flow cytometry analysis. Consistently, the percentages of M1 and M2 macrophages were not different between the two groups (**Figures 4L–O**). Together, these results indicate that CCL5 has no effect on the obesity-induced macrophage phenotype switch in eWAT. It has been reported that CCL5 promotes the migration of human adipose tissue macrophages and protects macrophages from apoptosis induced by free cholesterol (25, 26). We examined whether CCL5 is a chemokine for macrophages and protects macrophages from apoptosis *in vitro*. As shown in **Figure S7A**, CCL5 promoted chemotaxis of peritoneal macrophages. The chemotactic effect of CCL5 on macrophages would promote a decrease of macrophage in adipose tissue in CCL5 KO mice. However, flow cytometry analysis showed that CCL5 increased the percentage of apoptosis of macrophages induced by LPS and palmitate (**Figures S7B–E**). In addition, there were less apoptotic macrophages in adipose tissue in CCL5 KO mice than WT mice (**Figures S7F**). This pro-apoptotic effect of CCL5 on macrophage would promote an increase of macrophage in adipose tissue in CCL5 KO mice. Therefore, in obese CCL5 KO mice, the content of macrophage in eWAT was not altered, which may be attributed to the balance between reduced recruitment and apoptosis of macrophage in eWAT.

### 3.5 CCL5 deficiency increases HFD-induced lipid accumulation in the liver

**Figure 5:**
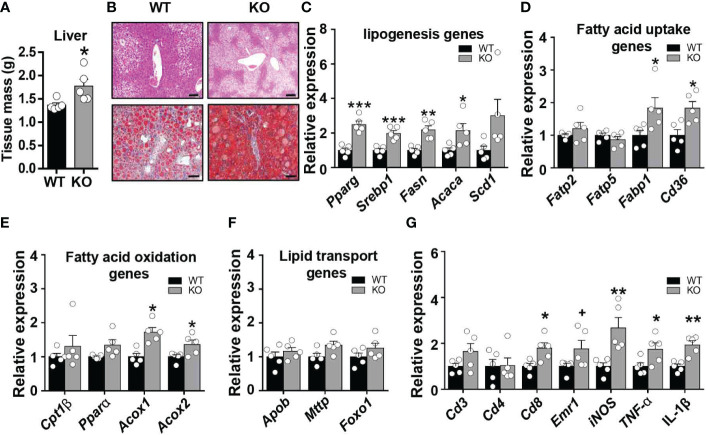
CCL5 deficiency promotes HFD-induced lipid accumulation in liver. **(A)** Weight of liver from WT and CCL5 KO mice after 16 weeks HFD. (n = 5 mice per group). **(B)** Representative H&E (Scale bar: 100 μm) and Oil red O (Scale bar: 50 μm) stained section of liver from WT and CCL5 KO mice after 16 weeks HFD. **(C)** mRNA expression of lipogenesis genes in liver from WT and CCL5 KO mice after 16 weeks HFD. (n = 5 mice per group). **(D)** mRNA expression of fatty acid uptake genes in liver from WT and CCL5 KO mice after 16 weeks HFD. (n = 5 mice per group). **(E)** mRNA expression of fatty acid oxidation genes in liver from WT and CCL5 KO mice after 16 weeks HFD. (n = 5 mice per group). **(F)** mRNA expression of lipid transport genes in liver from WT and CCL5 KO mice after 16 weeks HFD. (n = 5 mice per group). **(G)** mRNA expression of immune cell markers and cytokines in the liver from WT and CCL5 KO mice after 16 weeks HFD. (n = 5 mice per group). Data are mean ± s.e.m. ^+^ *p*<0.1, *p<0.05, **p<0.01, ***p<0.001 by unpaired Student’s t-test.

A marked impairment in insulin responsiveness was observed in the liver of obese CCL5 KO mice (**Figure 3G**). Fat accumulation in the liver is strongly associated with hepatic insulin resistance in obesity (27). We, therefore, examined the obesity-induced hepatic steatosis in WT and CCL5 KO mice. HFD feeding induced higher liver weight in CCL5 KO mice than in WT mice (**Figure 5A**). Moreover, a notable increase in lipid deposition was observed in the liver of obese CCL5 KO mice compared with WT mice, as demonstrated by H&E and Oil Red O staining (**Figure 5B**). To further explore the phenotype of hepatic steatosis in obese CCL5 KO mice, the key genes regulating processes of lipid metabolism were characterized by qRT-PCR. The mRNA levels of lipogenesis genes (*Pparγ*, *Srebp1c*, *Fasn*, and *Acaca*) and fatty acid uptake genes (*Fabp1* and *Cd36*) in the liver were increased in obese CCL5 KO mice than in obese WT mice (**Figures 5C, D**). While, the mRNA levels of fatty acid oxidation genes (*Acox1* and *Acox2*) increased slightly in obese CCL5 KO mice (**Figure 5E**), and the mRNA levels of fatty acid transport genes were not different between the two groups (**Figure 5F**). These data indicate that CCL5 deficiency promotes obesity-induced hepatic lipid accumulation. This is most likely the result of increased hepatic lipogenesis and lipid uptake rather than decreased lipid oxidation. Furthermore, the mRNA expression levels of the immune cell markers and proinflammatory cytokines in the livers of obese CCL5 KO and WT mice were examined. The mRNA levels of *Cd8*, *iNOS*, *TNF-α*, and *IL-1β* were increased in obese CCL5 KO mice than in obese WT mice (**Figure 5G**), indicating that CCL5 deficiency also exacerbates liver inflammation in obesity. Together, these data indicate that CCL5 deficiency aggravates obesity-induced liver injury. To further investigate whether CCL5 directly acts on lipid metabolism in hepatocytes, we examined the effect of CCL5 on hepatocyte steatosis *in vitro*. Oil O red staining and quantitation of lipid loading experiment showed that CCL5 decreased palmitate-induced lipid accumulation in hepatocytes (**Figure S8A, B**). Moreover, CCL5 reduced mRNA expression of genes involved in lipogenesis and fatty acid uptake in primary hepatocytes (**Figures S8C, D**). These results indicated that CCL5 has direct effects on modulating lipid metabolism in hepatocytes.

## 4 Discussion

Obesity-induced inflammation is closely associated with insulin resistance and type 2 diabetes (6). Targeting the key regulators involved in the recruitment and activation of proinflammatory immunocytes could be a potential therapeutic approach for insulin resistance and type 2 diabetes. CCL5 acts as a vital factor to trigger T lymphocytes and monocyte/macrophages chemotaxis and activation in chronic inflammatory diseases (28–30). However, the role of CCL5 in obesity-induced adipose tissue inflammation remains obscure. Our study detect the expression of CCL5 in white adipose tissue and the function of CCL5 in metabolic regulation in lean and obese mice. First, the expression of CCL5 is increased in eWAT of HFD-induced obesity, particularly in CD8^+^ T cells within eWAT. Second, CCL5 deficiency enhances glucose tolerance in lean mice but deteriorate insulin resistance by upregulating T cell-mediated adipose inflammation in obese mice. Finally, CCL5 deficiency increases the expression of chemokines, which could trigger T cells chemotaxis in adipose tissue of obese mice.

Our results demonstrated elevated expression of CCL5 in the eWAT of obese mice, which is consistent with previous studies (12). Adipose tissue is composed of adipocytes and SVF. Wu et al. (12) identified markedly higher mRNA expression levels of CCL5 in SVF than in adipocytes. Since SVF includes many kinds of cells, such as ASCs, T cells, and macrophages, it was unclear which cell type is the primary source of CCL5 in obese adipose tissue. We identified that CD8^+^ T cells are the major cellular sources responsible for upregulation of CCL5 by obesity in eWAT. Nishimura et al. (23) demonstrated that infiltration of CD8^+^ T cells is an early event in adipose tissue inflammation induced by obesity. Additionally, the accumulation of CD8^+^ T cells enhanced the infiltration of macrophages (23). As CCL5 could promote macrophage recruitment (31), CCL5 may be an essential chemokine secreted by CD8^+^ T cells to enhance the recruitment of macrophages during the development of obesity-induced adipose tissue inflammation.

In our study, CCL5 deficiency did not affect insulin sensitivity under lean conditions. However, Chou et al. (32) found impaired insulin sensitivity in lean CCL5 deficient mice. This divergency likely stems from differences in insulin dose applied to ITT experiments. We used 0.45 U/kg of insulin to treat 5-month-old mice (body weight 25-30 g). In their mouse studies, Chou et al. (32) used 0.75 U/kg of insulin to treat 3-4-month-old mice (body weight 20-30 g). Too high an insulin dose may cause a counter-regulatory response to prevent hypoglycaemia, thus inducing ITT becomes a compound test of insulin sensitivity and counter-regulatory response (33). So, in Chou’s experiment, the result of ITTs may be related to the high insulin dose. The role of CCL5 in glucose metabolism remains under debate. Liu et al. (17) identified that CCL5 could stimulate insulin secretion *via* GPR75 in beta cells to improve mouse glucose tolerance. However, Pais et al. (21) demonstrated that CCL5 impaired glucose-induced insulin secretion by reducing the secretion of GLP1 and GLP2. Consistent with Pais et al., we found that CCL5 deficiency enhanced glucose tolerance by promoting insulin secretion under lean conditions. In addition, CCL5 can also downregulate islet beta cell proliferation by directly inhibiting islet beta cell proliferation and the GLP-1-induced proliferation of islet beta cell. This discrepancy may stem from that Liu et al. studied the role of CCL5 in glucose tolerance by a gain-of-function mice model through intraperitoneal injection of CCL5. In contrast, we analyzed by constructing a loss-of-function mice model.

In obesity, chronic adipose inflammation is a crucial contributor to impaired insulin sensitivity (4). CCL5 KO mice were more insulin resistant than WT mice under obese conditions, which was consistent with worse adipose inflammation in them. CCL5 could recruit M2 macrophages and increase the ratio of M2/M1 in the progression of hepatocellular carcinoma and osteogenesis (34, 35). However, only a slightly increased expression of M2 marker genes was observed in adipose tissue of CCL5 KO mice fed HFD, suggesting that macrophages may be not responsible for the exacerbated adipose inflammation. In contrast, a significant increased T cell accumulation was observed in obese CCL5 KO mice. Therefore, it is likely that the worse adipose inflammation in obese CCL5 KO mice is dependent on enhanced T cell accumulation in adipose tissue. Since CCL5 is a T cell chemokine, we did not expect an increased T cell accumulation in obese CCL5 KO mice. Chen et al. reported that CCL5 deficiency could compensatorily activate the CXCL1-CXCR2 axis in neutrophils to enhance their infiltration and liver injury in hepatitis (24). In our study, many T cell chemokines, including CCL2, CCL7, CCL8, CXCL1, and CXCL10 (30, 36, 37), were dramatically increased in the adipose tissue of obese CCL5 KO mice. CCL5 deficiency may compensatorily induce the production of other chemokines, which may account for the increased accumulation of T cells in the adipose tissue of obese CCL5 KO mice. However, the mechanism of this compensatory regulation needs to be further investigated.

CCL5 KO mice showed more severe lipid accumulation in liver after HFD feeding than WT mice. Since the processes of hepatic lipogenesis and lipid uptake were altered in obese CCL5 deficient mice, these processes may account for severe hepatic steatosis caused by CCL5 deficiency. As adipose tissue dysfunction could promote the progression of hepatic steatosis due to insulin resistance and proinflammatory adipokines release (38), the heaven hepatic steatosis may ascribe to severe adipose tissue dysfunction in obese CCL5 KO mice. Park et al. reported that increased pro-inflammatory chemokines and cytokines induced by CCR5 deficiency may cause hepatic injury (39), raising the possibility that CCL5 deficiency may lead to overexpression of pro-inflammatory factors and tissue injury in the liver. Indeed, the mRNA expression levels of inflammatory markers were increased in the liver of CCL5 deficient mice. However, the mechanisms underlying immunocytes contributed to the obesity-induced nonalcoholic steatohepatitis in CCL5 deficient mice still need to be clarified. In addition, CCL5 can directly inhibit lipogenesis in primary hepatocytes. Therefore, the increased hepatic steatosis in obese CCL5 KO mice may be caused not only by insulin resistance and inflammation but also by the loss of the direct influence of CCL5 on hepatocyte lipogenesis. Insulin resistance is associated with hepatic steatosis. However, it is unclear whether hepatic steatosis is a cause or a consequence of insulin resistance (27). Therefore, whether hepatic steatosis triggers insulin resistance in obese CCL5 KO mice remains to be studied. A limitation of our study is that it was performed in male mice only. Further studies should be carried out to evaluate the metabolic effects of CCL5 in female mice and other animal species.

## Data availability statement

The original contributions presented in the study are included in the article/**Supplementary Material**. Further inquiries can be directed to the corresponding author.

## Ethics statement

The animal study was reviewed and approved by the Department of Laboratory Animal Science, Central South University.

## Author contributions

HuZ and XL contributed to the study design, acquisition of data, analysis, and interpretation of results, as well as drafting and revision of the manuscript. QZ, HaZ, JS, WH, XS, YD, DW, and YX contributed to the acquisition of the data and revision of the manuscript. TD contributed to the conception and design of the study, analysis of results and the revision of the manuscript. All authors gave their approval for the final manuscript to be published.
