# Supplementary material for: Metabolic effects of CCL5 deficiency in lean and obese mice
Source: Front Immunol. 2023 Jan 13;13:1059687. doi: 10.3389/fimmu.2022.1059687 (PMC9880418; doi:10.3389/fimmu.2022.1059687)
Supplement: Supplementary file 1 [file DataSheet_1.docx]

Supplementary Material

# Supplementary Figures and Tables

## Supplementary Figures


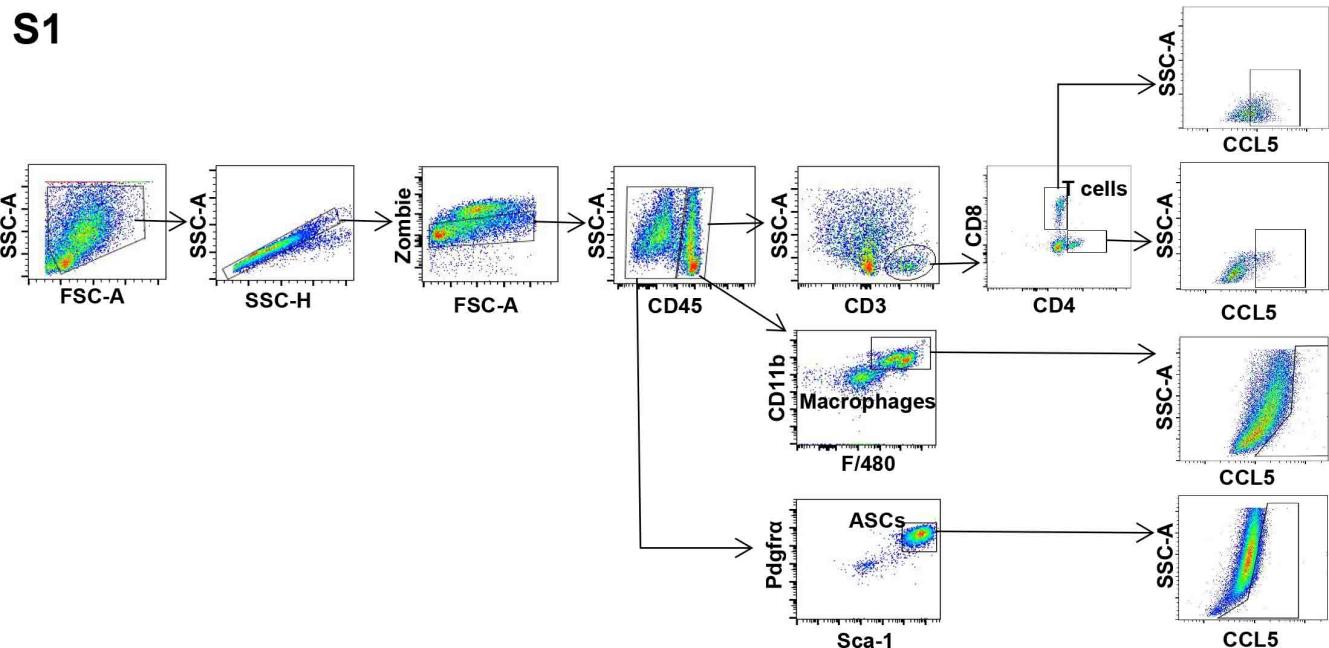


## Supplementary Figure 1. Sequential gating strategy for analysis of CCL5 expression in T cells, macrophages, and ASCs. Flow cytometry plots of SVF isolated from eWAT of ND-fed and 16-week HFD-fed mice. After excluding cell debris and dead cells by FSC/SSC parameters and Zombie staining, CD4^+^ T cells were identified as CD45^+^CD3^+^CD4^+^ cells, CD8^+^ T cells were identified as CD45^+^CD3^+^CD8^+^ cells, Macrophages were identified as CD45^+^CD11b^+^ F4/80^+^ cells, ASCs were identified as CD45^-^Pdgfra^+^Sca-1^+^cells. CCL5^+^ cells were gated by unstained control.


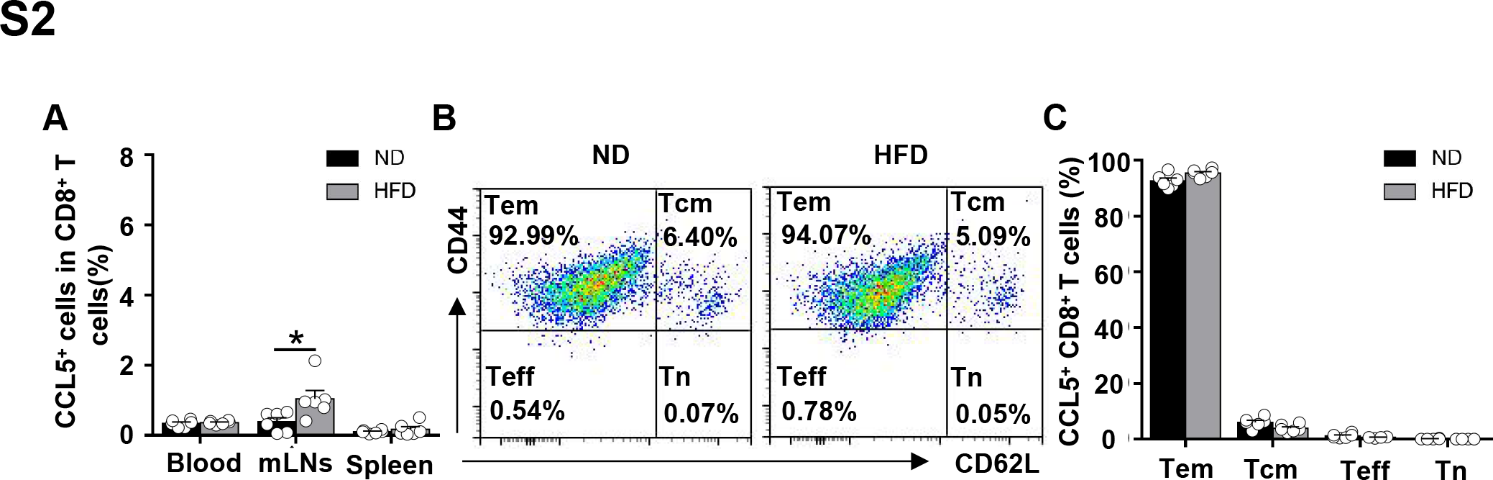


**Supplementary Figure 2** CCL5 is upregulated in CD8^+^ T effector memory subset in eWAT in obese mice. **(A)** Quantification of CCL5^+^ cells in CD8^+^ T cells of blood, mesenteric lymph nodes (mLNs), and spleen in C57BL/6J mice fed the ND or HFD by flow cytometry analysis. (n = 6 mice per group). **(B)** Representative flow cytometry plots of CCL5^+^ CD8^+^ T cells subsets (T naive—Tn, T effector memory—Tem, T central memory—Tcm, T effector—Teff). **(C)** Quantification of CD8^+^ T cells subsets in CCL5^+^ CD8^+^ T cells. Data are mean ± s.e.m.**p*<0.05 by unpaired Student’s *t*-test.


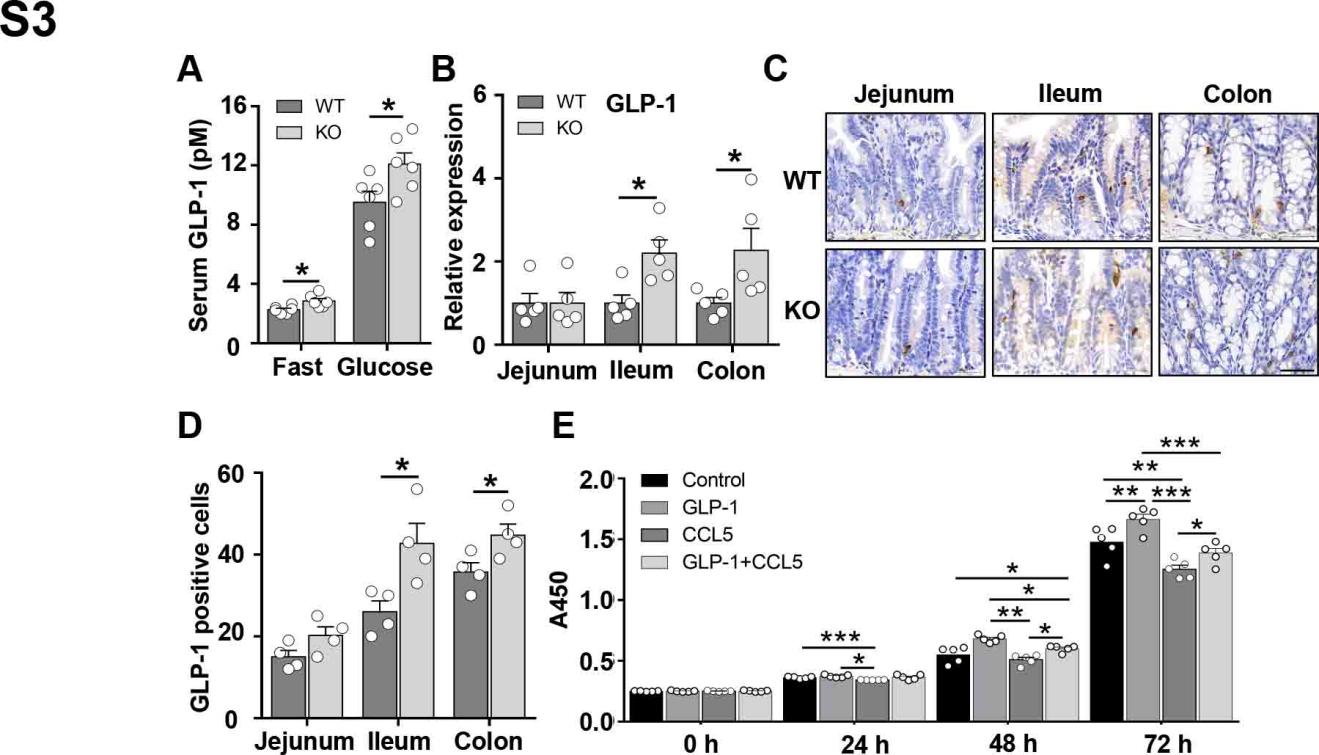


**Supplementary Figure 3** CCL5 down-regulates GLP-1 expression and function. **(A)** Serum GLP-1 levels of ND-fed mice after 6 h fasting (fast) or 15 min after 2g/kg glucose gavage (glucose). (n = 5 mice per group). **(B)** mRNA expression of GLP-1 in jejunum, ileum, and colon of WT and CCL5 KO mice fed an ND. (n = 5 mice per group). **(C)** Representative immuohistochemical staining of GLP-1 in jejunum, ileum, and colon of WT and CCL5 KO mice fed an ND. Scale bar: 50 μm. **(D)** Quantification of images for GLP-1 in jejunum, ileum, and colon of WT and CCL5 KO mice fed an ND. (n = 4 mice per group). **(E)** Proliferation of Min6 cells treated with or without CCL5 (100 ng/ml) or GLP-1 (100 ng/ml). Data in (A-B and D) are mean ± s.e.m.*p<0.05 by unpaired Student’s t-test. Data in (E) are mean ± s.e.m.**p*<0.05,***p*<0.01, ****p*<0.001 by two-way ANOVA.


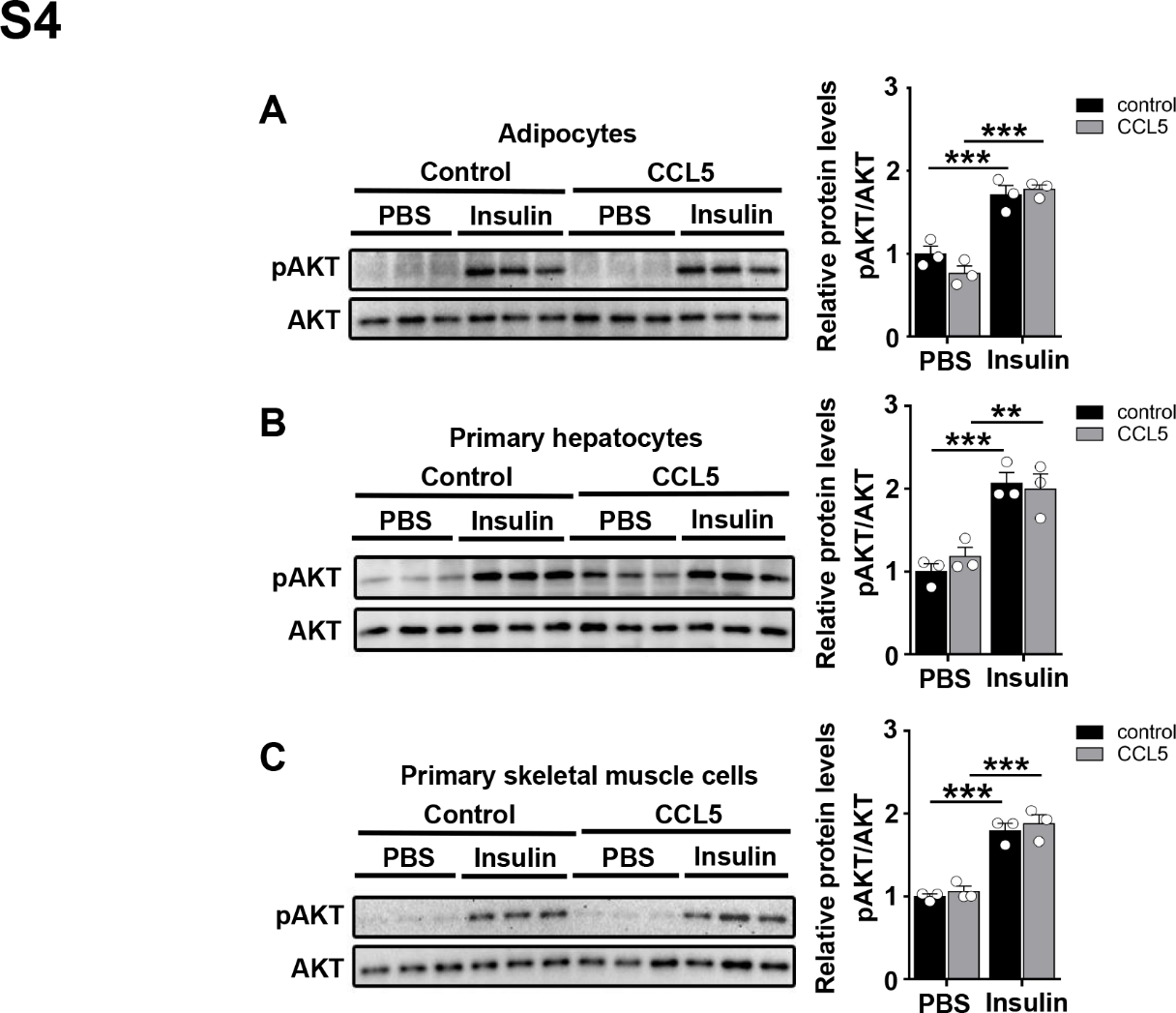


**Supplementary Figure 4** CCL5 has no effect on insulin signaling in insulin target cells. **(A-C)** Western blot (left) and quantification (right) of p-AKT and AKT in the adipocytes (A), primary hepatocytes (B), and primary skeletal muscle cells (C) treated with or without CCL5 (100 ng/ml). (n = 6 per group). Data are mean ± s.e.m.***p*<0.01, ****p*<0.001 by two-way ANOVA.


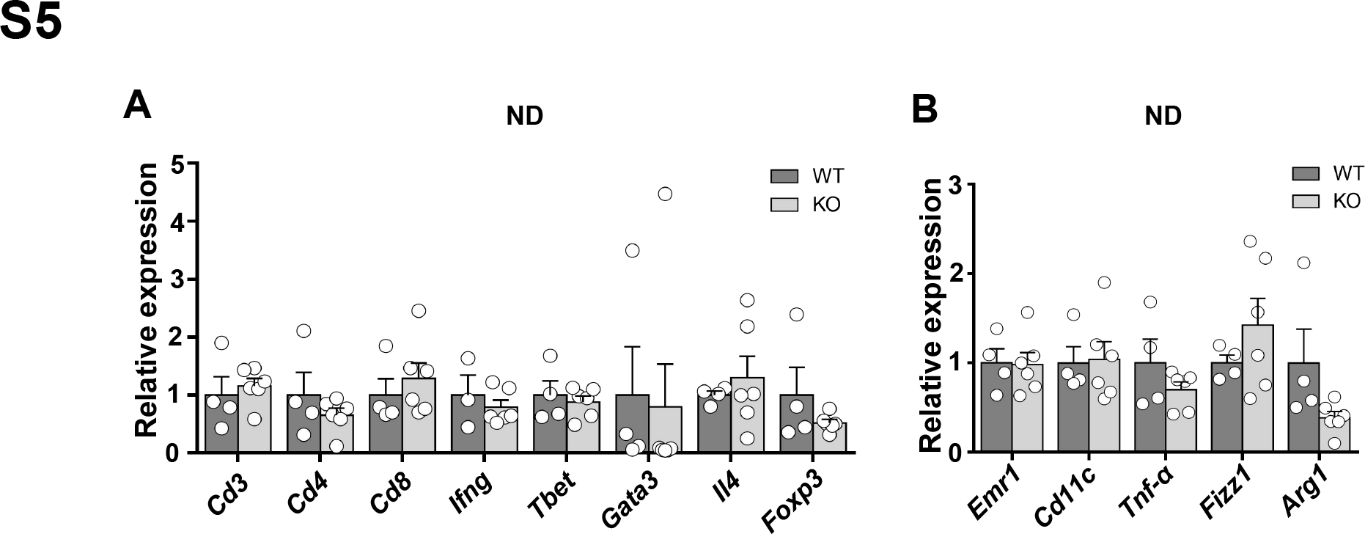


**Supplementary Figure 5** CCL5 deficiency has no effect on immune cell marker genes expression in eWAT of ND-fed mice.  **(A)** mRNA expression of T cell marker genes in eWAT of WT and CCL5 KO mice fed an ND. (n=4-6 mice per group). **(B)** mRNA expression of macrophage marker genes in eWAT of WT and CCL5 KO mice fed an ND. (n = 4-6 mice per group). Data are mean ± s.e.m. The two group differences were analyzed with unpaired Student’s *t*-test.


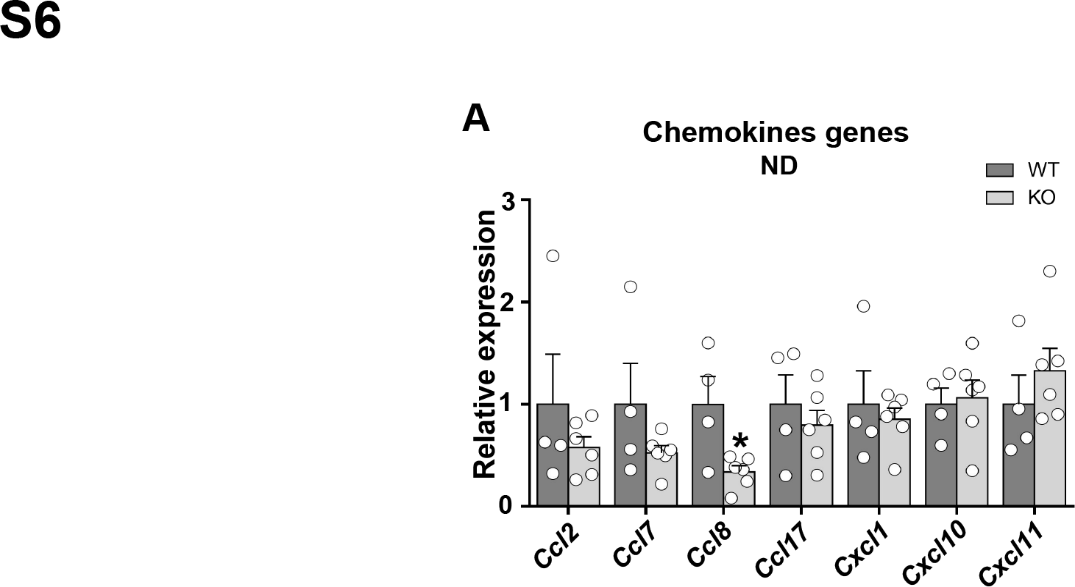


**Supplementary Figure 6** CCL5 deficiency has no effect on chemokines expression in eWAT of lean mice. **(A)** mRNA expression of chemokine genes in eWAT of WT and CCL5 KO mice fed an ND. (n=4-6 mice per group). Data are mean ± s.e.m.**p*<0.05 by unpaired Student’s *t*-test.


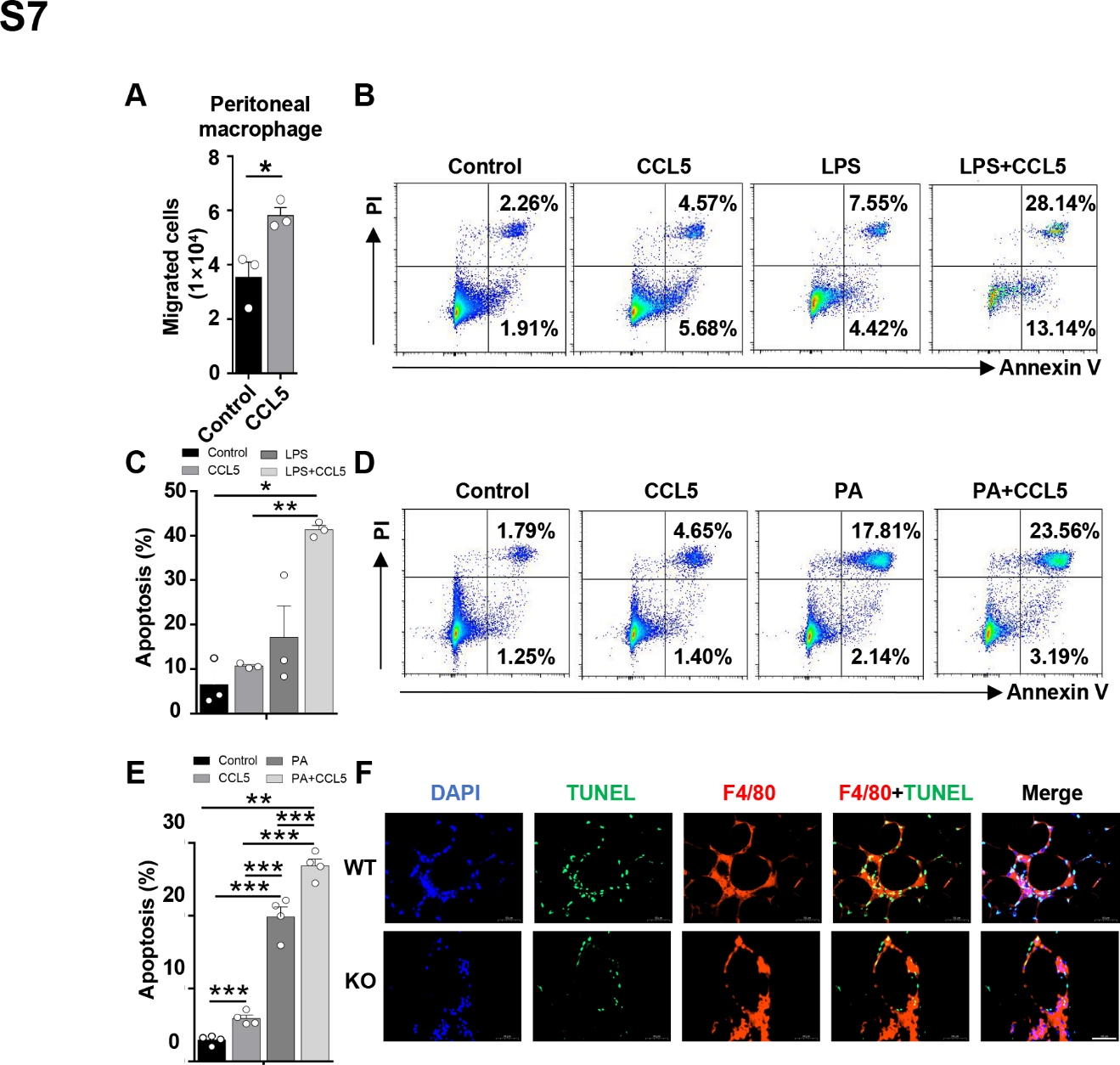


**Supplementary Figure 7** CCL5 promoted the migration and apoptosis of macrophages. **(A)** Effects of CCL5 on Peritoneal macrophage chemotaxis. **(B-C)** Representative flow cytometry plots (B) and quantification (C) of apoptotic Raw264.7 treated with or without LPS (1 μg/ml) and CCL5 (100 ng/ml). **(D-E)** Representative flow cytometry plots (D) and quantification (E) of apoptotic Raw264.7 treated with or without PA (300 μM) and CCL5 (100 ng/ml). **(F)** Representative immunofluorescent staining of F4/80 and TUNEL assay in the eWAT from WT and KO mice fed a HFD. Scale bar: 50 μm. Data in (A) are mean ± s.e.m.**p*<0.05, ****p*<0.001 by unpaired Student’s t-test. Data in (C and E) are mean ± s.e.m.**p*<0.05, ***p*<0.01, ****p*<0.001 by two-way ANOVA.


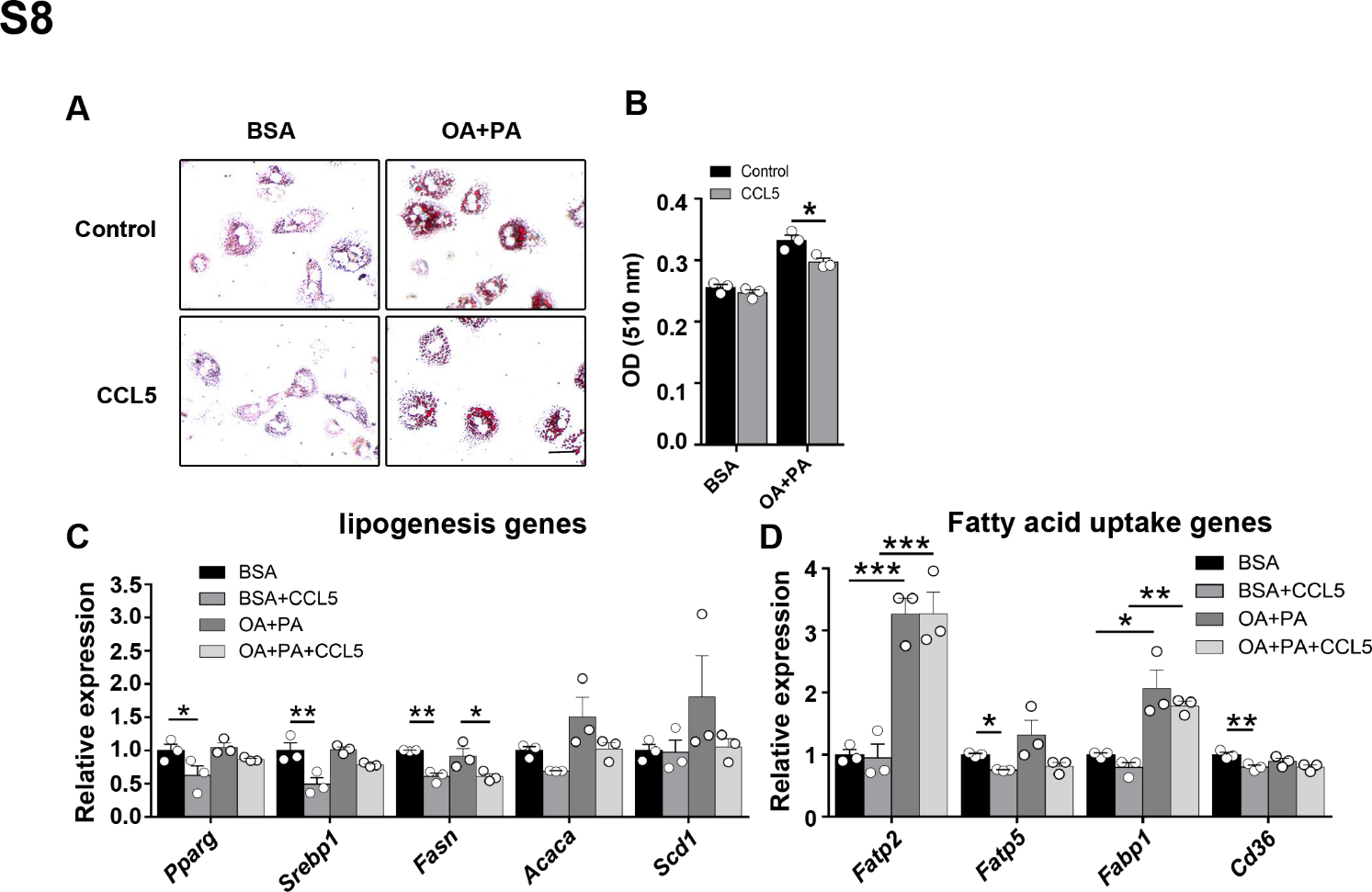


**Supplementary Figure 8** CCL5 inhibits lipid accumulation in primary hepatocytes. **(A)** Representative Oil red O staining of primary hepatocytes with or without CCL5 (100 ng/ml). Scale bar: 50 μm. **(B)** Quantitative total lipid by measuring the 510 nm absorbance after eluting Oil red O dye with isopropanol. **(C-D)** mRNA expression of lipogenesis genes (C), fatty acid uptake genes (D) in primary hepatocytes with or without CCL5 (100 ng/ml). Data in (B) are mean ± s.e.m.**p*<0.05 by unpaired Student’s t-test. Data in (C-D) are mean ± s.e.m.^+^*p*<0.1, **p*<0.05, ***p*<0.01, ****p*<0.001 by two-way ANOVA.

# Supplementary Tables

## Supplementary Table 1. Antibodies used in FACS analysis

| **Antibody** | **Company** |  |
| --- | --- | --- |
| CD45-FITC | Biolegend | Cat# 103108; RRID: AB_312973 |
| CD45-Percp/Cy5.5 | Biolegend | Cat# 103132; RRID: AB_893340 |
| CD3-Alexa Fluor 700 | Biolegend | Cat# 100216; RRID: AB_493697 |
| CD3-Percp/Cy5.5 | Biolegend | Cat# 100218; RRID: AB_1595597 |
| CD3-APC | Biolegend | Cat# 100236; RRID: AB_2561456 |
| CD4-PerCP | Biolegend | Cat# 100537; RRID: AB_893331 |
| CD4-APC/Cy7 | Biolegend | Cat# 100414; RRID: AB_312699 |
| CD4-PE | Biolegend | Cat# 100408; RRID: AB_312693 |
| CD8-BB515 | BD Bioscience | Cat# 564459; RRID: AB_2738801 |
| CD8-PE/Cy7 | Biolegend | Cat# 100722; RRID: AB_312761 |
| CD8-APC | Biolegend | Cat# 100712; RRID: AB_312751 |
| CD44-APC | Biolegend | Cat# 103012; RRID: AB_312963 |
| CD62L-APC/CY7 | Biolegend | Cat# 104428; RRID: AB_830799 |
| CCL5-PE | Biolegend | Cat# 149103; RRID: AB_2564405 |
| F4/80-PE/Cy5 | Biolegend | Cat# 123111; RRID: AB_893494 |
| F4/80-PE/Cy7 | Biolegend | Cat# 123114; RRID: AB_893478 |
| F4/80-PE/Dazzle594 | Biolegend | Cat# 123146; RRID: AB_2564133 |
| Cd11b-Percp/Cy5.5 | Biolegend | Cat# 101228; RRID: AB_893232 |
| CD11c-PE | Biolegend | Cat# 117308; RRID: AB_313777 |
| CD206-APC | Biolegend | Cat# 141708; RRID: AB_10900231 |
| Sca1-APC/Cy7 | Biolegend | Cat# 108126; RRID: AB_10645327 |
| Pdgfra-PE/Cy7 | Biolegend | Cat# 135912; RRID: AB_2715974 |

## Supplementary Table 2. Primer sequence used for real-time PCR

| **Primer** | **Primer sequence** |
| --- | --- |
| β-actin | Forward: CTACCTCATGAAGATCCTGACC  Reverse: CACAGCTTCTCTTTGATGTCAC |
| 36B4 | Forward: CGTCCTCGTTGGAGTGACAT  Reverse: TAGTTGGACTTCCAGGTCGC |
| Acaca | Forward: TGGAGAGCCCCACACACA  Reverse: TGACAGACTGATCGCAGAGAAAG |
| Acox1 | Forward: GGGCACGGCTATTCTCACAG  Reverse: CATCAAGAACCTGGCCGTCT |
| Acox2 | Forward: ACGGTCCTGAACGCATTTATG  Reverse: TTGGCCCCATTTAGCAATCTG |
| Arg1 | Forward: CTCCAAGCCAAAGTCCTTAGAG  Reverse: AGGAGCTGTCATTAGGGACATC |
| Apob | Forward: ATTCGAGCACAGATGACCAG  Reverse: GTACCTTTCACCATCAGACTCC |
| Ccl2 | Forward: AGGTCCCTGTCATGCTTCTG  Reverse: TCTGGACCCATTCCTTCTTG |
| Ccl5 | Forward: GCCCACGTCAAGGAGTATTT  Reverse: CTTGAACCCACTTCTTCTCTGG |
| Ccl7 | Forward: ACAAAAGATCCCCAAGAGGAAT  Reverse: TCTTGAAGATAACAGCTTCCCA |
| Ccl8 | Forward: GAATCAACAATATCCAGTGCCC  Reverse: TTGAGACTTCTGGTCAAGGATC |
| Ccl17 | Forward: AGACCTTCACCTCAGCTTTTG  Reverse: CTTTGAAGTAATCCAGGCAGC |
| Ccr1 | Forward: ATCCTGTTGACGATTGACAGAT  Reverse: TGATGCCAAAAGTAACAGTTCG |
| Ccr3 | Forward: AATCAAGACTGTGGTTGAAAGC  Reverse: GAGGATCAACACAACCATCATG |
| Ccr5 | Forward: TCCAGCAAGACAATCCTGATC  Reverse: AACCATTCCTACTCCCAAGC |
| Cd3 | Forward: GAACCAGTGTAGAGTTGACGTG  Reverse: CCAGGTGCTTATCATGCTTCTG |
| Cd4 | Forward: CAGCATGGCAAAGGTGTATTAA  Reverse: GACTGAAGGTCACTTTGAACAC |
| Cd8 | Forward: AGTTAACAAGCATCTACTGGCT  Reverse: TCTTGTCCACACTTTCACCATA |
| Cd11c | Forward: TCATCACTGATGGGAGAAAACA  Reverse: CCCCAATTGCATAACGAATGAT |
| Cd36 | Forward: GCGACATGATTAATGGCACAG  Reverse: GATCCGAACACAGCGTAGATAG |
| Cpt1β | Forward: TGGCTACGGGGTCTCTTACA  Reverse: TGGGCGTTCGTCTCTGAAC |
| Cxcl1 | Forward: AACCGAAGTCATAGCCACAC  Reverse: CAGACGGTGCCATCAGAG |
| Cxcl10 | Forward: ATCATCCCTGCGAGCCTATCCT  Reverse: GACCTTTTTTGGCTAAACGCTTTC |
| Cxcl11 | Forward: ATGGCAGAGATCGAGAAAGC  Reverse: TGCATTATGAGGCGAGCTTG |
| Emr1 | Forward: TTTCAAATGGATCCAGAAGG  Reverse: CAGAAGGAAGCATAACCAAG |
| Fasn | Forward: AGAGACGTGTCACTCCTGGACTT  Reverse: GCTGCGGAAACTTCAGAAAAT |
| Fatp2 | Forward: ACATCTACTTCAACAGCGGAG  Reverse: ACAAAATCTACCAGTCCCACG |
| Fatp5 | Forward: ACGTCCTACCTCTGTACCATAC  Reverse: CAAGATCACTGTTACGCCATG |
| Fabp1 | Forward: TCTCCGGCAAGTACCAATTG  Reverse: TTGATGTCCTTCCCTTTCTGG |
| Fizz1 | Forward: GGAACTTCTTGCCAATCCAGC  Reverse: AAGCCACAAGCACACCCAGT |
| Foxo1 | Forward: ATGTGTTGCCCAACCAAAGC  Reverse: AGGACTTTTAAATGTAGCCTGCTC |
| Foxp3 | Forward: TTTCACCTATGCCACCCTTATC  Reverse: CATGCGAGTAAACCAATGGTAG |
| Gata3 | Forward: ATTACCACCTATCCGCCCTAT  Reverse: CGGTTCTGCCCATTCATTTTAT |
| Gpr75 | Forward: GAATATCAACCAGATGCCAATC  Reverse: GGAAATCCCAAGAGGAAGAC |
| GLP1 | Forward: ACGAGATGAGCACCATTCTG  Reverse: TGACTGGCACGAGATGTTG |
| Ifn-γ | Forward: CTTTGGACCCTCTGACTTGAG  Reverse: TCTTCCACATCTATGCCACTTG |
| IL4 | Forward: CGAATGTACCAGGAGCCATATC  Reverse: TCTCTGTGGTGTTCTTCGTTG |
| IL-1β | Forward: GAAATGCCACCTTTTGACAGTG  Reverse: TGGATGCTCTCATCAGGACAG |
| Mttp | Forward: TCCACATACAGCCTTGACATC  Reverse: TTAAGCCTTCCAGCCCTTG |
| iNOS | Forward: TCCTGGACATTACGACCCCT  Reverse: CTCTGAGGGCTGACACAAGG |
| Tbet | Forward: GATCACTCAGCTGAAAATCGAC  Reverse: AGGCTGTGAGATCATATCCTTG |
| Tnf-α | Forward: CCCTCCAGAAAAGACACCATG  Reverse: GTCTGGGCCATAGAACTGATG |
| Srebf1 | Forward: GGAGCCATGGATTGCACATT  Reverse: GGCCCGGGAAGTCACTGT |
| Scd1 | Forward: CATCATTCTCATGGTCCTGCT  Reverse: CCCAGTCGTACACGTCATTTT |
| Pparg | Forward: TCCAGCATTTCTGCTCCACA  Reverse: ACAGACTCGGCACTCAATGG |
| Pparα | Forward: CATTTCCCTGTTTGTGGCTG  Reverse: ATCTGGATGGTTGCTCTGC |
